# Mutational spectrum of *WTX*, *WT1*, *CTNNB1*, *APC* and *PLCG2* genes in Wilms tumor defined by massive parallel resequencing

**DOI:** 10.1186/1753-6561-6-S6-P52

**Published:** 2012-10-01

**Authors:** Bruna D Barros, Giovana T Torrezan, Elisa N Ferreira, Mariana Maschietto, Ana CV Krepíschi, Dirce M Carraro

**Affiliations:** 1 CIPE- Internationa! Center of Research and Training-A. C. Camargo Hospital, São Paulo, SP, Brazil; 2 MHCB Unit, UCL Institute of Child Health, London, UK

## Background

The identification of molecular alterations that trigger Wilms tumor (WT) development is crucial to understanding the tumorigenesis of this malignancy. Currently, it is estimated that *WTX* and *WT1* genomic losses together with *CTNNB1* point mutations occur in about 30% of WTs. However, the majority of cases remain without any identified driver mutation. Results from a previous study by our group pointed to *APC* and *PLCG2* as candidate genes altered in WT [1]. Given the advent of modern DNA sequencing technologies, it is now feasible to evaluate large genomic regions spanning complete genes (exons and introns), allowing the description of the mutation patterns occurring in tumor cells. Thus, the aim of this study was to identify point mutations and indels in the complete sequence of *APC*, *CTNNB1*, *WT1*, *WTX* and *PLCG2* genes in order to characterize both the exonic mutational spectrum and the intronic nucleotide substitution pattern.

## Material and methods

The complete genomic regions of the selected genes, spanning a total of 430 kb, were amplified by long-range PCR in 15 WTs and 3 non-neoplasic control samples, giving a total of 60 amplicons per sample (10 kb on average). The resulting amplicons were mixed at equimolar concentrations and, for each sample, the Ion PGM library preparation protocol was performed. The libraries of the 18 bar-coded samples were combined in four sequencing pools that were individually submitted to an Ion PGM™ Sequencer run on an Ion 316™ Chip. Point mutation and indels not present in the non-neoplasic controls were selected for capillary sequencing validation. The validated alterations were screened in an independent group of 39 WT and 96 normal control samples.

## Results

For the 15 tumors and 3 normal controls, we obtained 400,000 sequences per sample on average (350× mean target coverage). At the exonic regions, four out of the four identified missense alterations were validated. None of the 37 identified indels could be confirmed, probably due to indel errors generated by the Ion PGM platform [2]. Three of the four missense alterations were not identified in any of the 99 controls, and therefore were classified as possibly associated with WT: two at *APC* (Ile2541Val and Met1413Val) and one at *PLCG2* (Asn946Ser). No point mutation was observed for *WT1* and *CTNNB1* genes. In the intronic regions, the somatic substitution pattern was evaluated by classifying the tumor's substitutions in two groups: single nucleotide polymorphisms (SNPs) (alterations also present in normal samples, dbSNP and 1000genomes) and somatic substitutions (remaining alterations). A general trend in the somatic base substitution pattern was observed, with the tumors presenting an over-representation of G:C>A:T changes (*P* >0.001) and a reduction of A:T>G:C changes (*P* >0.001) when compared to the SNP variants. Regarding the frequency of the other classes of somatic substitutions, a wide variation was observed among the different tumors.

## Conclusions

This study provides insights into the spectrum of WT substitution mutations occurring in exonic and intronic regions of five previously associated genes, revealing a low frequency of point mutations in the coding sequence of these genes and a general over-representation of somatic G:C>A:T transitions.
